# Contextualization Procedure and Modeling of Monocyte Specific TLR Signaling

**DOI:** 10.1371/journal.pone.0049978

**Published:** 2012-12-06

**Authors:** Maike K. Aurich, Ines Thiele

**Affiliations:** 1 Center for Systems Biology, University of Iceland, Reykjavik, Iceland; 2 Faculty of Industrial Engineering, Mechanical Engineering and Computer Science, University of Iceland, Reykjavik, Iceland; Ajou University, Republic of Korea

## Abstract

Innate immunity is the first line of defense against invasion of pathogens. Toll-like receptor (TLR) signaling is involved in a variety of human diseases extending far beyond immune system–related diseases, affecting a number of different tissues and cell-types. Computational models often do not account for cell-type specific differences in signaling networks. Investigation of these differences and its phenotypic implications could increase understanding of cell signaling and processes such as inflammation. The wealth of knowledge for TLR signaling has been recently summarized in a stoichiometric signaling network applicable for constraint-based modeling and analysis (COBRA). COBRA methods have been applied to investigate tissue-specific metabolism using omics data integration. Comparable approaches have not been conducted using signaling networks. In this study, we present ihsTLRv2, an updated TLR signaling network accounting for the association of 314 genes with 558 network reactions. We present a mapping procedure for transcriptomic data onto signaling networks and demonstrate the generation of a monocyte-specific TLR network. The generated monocyte network is characterized through expression of a specific set of isozymes rather than reduction of pathway contents. While further tailoring the network to a specific stimulation condition, we observed that the quantitative changes in gene expression due to LPS stimulation affected the tightly connected set of genes. Differential expression influenced about one third of the entire TLR signaling network, in particular, NF-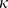
B activation. Thus, a cell-type and condition-specific signaling network can provide functional insight into signaling cascades. Furthermore, we demonstrate the energy dependence of TLR signaling pathways in monocytes.

## Introduction

Toll-like receptors (TLRs) play a major role in innate immunity for sensing pathogens and inducing innate immune response [1]. Each TLR specifically recognizes one or more exogenous and endogenous ligands. Exogenous ligands are highly conserved microbial associated molecular pattern, e.g., CpG sequences within DNA or lipopolysaccharide (LPS), a cell wall component of gram-negative bacteria. Upon stimulation downstream pathways and transcription factors (TF) are activated which modify gene expression and protein levels and induce production of pro-inflammatory cytokines and chemokines, amongst others. Human cells express up to ten TLRs [1]. LPS induces specifically TLR4 signaling pathways [1].

Disturbance of TLR signaling is thought to play a role in chronic inflammatory diseases affecting cells of the gastrointestinal tract, the central nervous system, kidneys, skin, lungs, and joints [2]. TLRs also seem to be involved in both inhibiting and promoting cancer [3]. TLRs expression has been confirmed for a large number of human tissues, yet sets of expressed TLRs vary [4]–[6]. Activation of differing downstream pathways has been suggested as response to viruses, TLR7, and TLR8 agonists in distinct monocytes subsets [7]. Differences and similarities in the expression of isoforms, TLRs and downstream pathways of the TLR network of different cells can have important implications for the design of therapeutical approaches. Drugs targeting TLR signaling pathways have considerable therapeutic potential in inflammatory diseases and cancer [8].

Monocytes are essential for the inflammatory response to microbial pathogens [9]. Blood circulating monocytes migrate into tissues and differentiate into a range of tissue macrophages and dendritic cells. However, monocytes themselves are involved in the defense against pathogens as they possess an extensive set of pathogen receptors and produce large quantities of effector molecules [9], [10]. Aberrant TLR signaling in the monocyte/macrophage cell lineage has been implicated in chronic inflammatory and auto-inflammatory diseases [11]. However, the reason for this increase in IL-1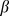 secretion for some of these diseases remains unknown [11]. Taken together, understanding TLR signaling at cell-type and tissue specific resolution seems to be of major importance for unraveling mechanisms underlying disease development and progression.

Signaling networks comprise a complex meshwork of multiple pathways, feedback loops, and cross-talk. Such complex networks may be best investigated using computational approaches. Constraint-based modeling and analysis (COBRA) techniques facilitate investigation of large-scale biological networks without depending on detailed kinetic and concentration information [12]. Instead, COBRA relies on physical-chemical constraints. A requirement for constraint-based modeling is a genome-scale reconstruction, which is subsequently converted into mathematical format. The protocols for biochemical network generation are well established [13] and tools to interrogate the model are freely available [14]. These networks are applied to study metabolism under various conditions, yet in multi-cellular organisms, challenged by the fact that individual cell-types are capable of only a limited range of metabolic functions. Hence, automated procedures have been developed that aim to tailor global genome-scale reconstructions tissue- and cell-type specific, based on ‘omics’ data sets [15]–[17]. COBRA procedures have also been applied to study successfully other cellular processes, including transcription and translation [18], [19], transcriptional regulation [20], [21], and signaling networks [22]–[25].

The published generic TLR signaling network, ihsTLRv1 [24], represents a stoichiometric, predictive model comprising of 963 reactions and 781 proteins. It includes the input receptors TLR1-11, NOD1, NOD2, and Interleukin-1 receptor 1 (IL1R1). These receptors are connected to up to six outputs, ROS, CREB, AP-1, IRF-7, IRF-3, and NF-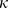
B (Table 1) through an extensive set of kinases and phosphatases. Due to its coverage, it is ideal to investigate TLR signaling pathways on a broader scale and to use it as context template for gene expression data sets. However, no gene identifiers and no gene-reaction associations were included, such that mapping of gene expression data and analysis of TLR signaling in cell-type or disease specific context, in analogy to applications of metabolic networks, was not possible so far. Tissue specific differences in the cell response to environmental stimuli have been recognized as major challenge in cell signaling, yet many models of signaling pathways neglect these cell-type specific differences [26].

**Table 1:** Inputs and outputs covered by generic (ihsTLRv2) and monocyte specific (hMonoTLR & hMonoTLR_LPS) TLR signaling models.

			Outputs						
Ligand Abbrev.	Ligand name	Receptor type activated	NF- 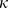 B	CREB	AP-1	ROS	IRF-7*	IRF-3	First model absent
26dap-LL	diaminopimelic acid	NOD1	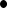						
ALPS	atypical lipopolysaccharide	TLR2	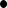	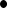	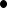	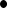			
BDFN2	beta defensin 2	TLR4	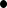	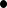	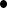	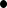	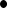	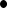	
BPM	bropirimine	TLR7	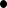	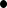	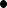	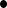	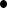		
CPGCIGC	CpG chromatic IgG2a complexs	TLR9	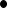	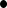	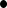	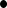	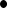		
CSGA	CsgA	TLR2	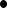	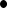	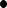	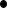			
DCLDLPP	diacetylated lipopeptides	TLR2/6	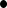	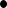	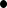	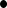			hMonoTLR_LPS
DCLLPP	diacyl lipopeptides	TLR2/6	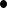	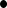	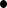	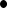			hMonoTLR_LPS
DSRNA	double stranded RNA	TLR3	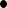			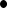	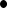	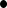	hMonoTLR
ENVP	envelope protein	TLR4	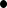	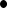	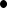	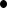	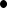	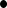	
FBNG	fibrinogen	TLR4	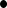	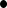	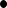	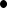	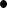	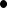	
FLGN	flagellin	TLR5	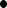	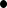	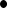	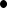	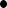		
FUSP	fusion protein	TLR4	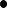	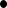	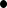	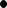	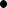	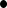	
GCSPL	glycoinositol phospholipids	TLR2	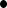	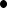	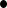	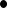			
GLC	glycolipids	TLR2	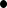	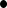	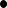	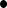			
HSP60	heat shock protein (60 kDa)	TLR4	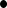	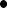	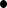	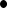	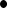	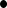	
HSP70	heat shock protein (70 kDa)	TLR2, TLR4	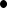	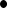	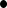	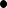			
IMQ	imidazoquinoline	TLR7, TLR8	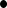	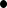	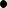	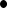	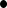		
LAM	lipoarabinomannan	TLR2	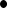	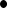	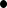	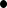			
LP	lipoprotein	TLR2	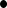	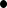	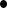	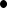			
LPPS	lipopeptides	TLR2	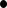	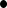	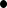	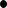			
LPS_HS	lipopolysaccharide (Homo sapiens)	TLR2, TLR4	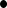	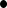	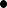	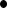			
LTA	lipoteichoic acid	TLR2/6, TLR2	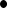	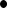	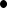	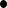			hMonoTLR_LPS
LXR	loxoribine	TLR7	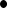	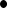	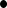	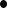	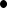		
MRAP	mannuronic acid polymer	TLR2, TLR4	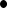	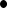	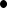	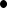			
MRDP	muramyl dipeptide	NOD2	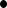						
MRNA	mRNA	TLR3	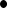			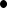	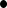	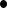	
OLSCHYA	oligosaccharides of hyaluronic acid	TLR4	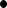	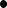	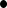	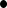	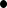	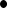	
OMPA	outer membrane protein A	TLR2	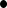	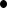	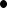	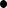			
OSPALP	outer surface protein A	TLR2/6	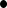	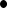	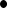	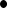			hMonoTLR_LPS
IL1A	IL-1A	IL1R1	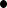	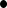	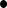	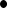			
IL1B	IL-1B	IL1R1	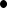	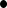	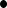	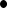			
PRNS	porins	TLR2	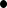	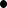	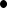	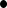			
PSCHPS	polysaccharide fragment of heparan sulphate	TLR4	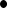	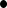	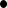	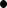	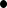	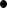	
PSM	phenol-soluble modulin	TLR2/6, TLR2	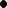	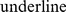	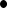	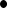			hMonoTLR_LPS
PTG_HS	peptidoglycan (Homo sapiens)	TLR2	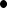	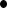	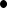	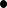			
SF	soluble factors	TLR1/2	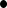	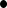	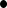	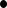			
SSRNA	single stranded RNA	TLR7, TLR8	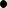	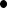	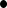	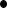	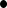		
STF	soluble tuberculosis factor	TLR2/6	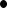	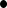	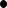	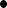			hMonoTLR_LPS
T3RFBN	type III repeat extra domain A of fibronectin	TLR4	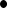	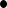	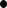	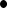	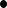	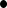	
TCLDLPP	triacetylated lipoproteins	TLR1/2	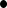	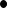	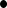	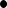			
TLRL1/10	TLR1/10 ligand	TLR1/10	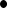	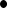	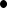	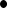			hMonoTLR
TLRL10	TLR10 ligand	TLR10	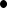	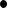	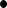	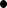			hMonoTLR
TLRL2/10	TLR2/10 ligand	TLR2/10	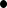	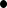	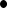	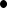			hMonoTLR
TXL	taxol	TLR4	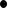	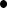	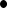	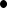	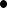	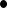	
UMLCPGD	unmethylated CpG DNA	TLR9	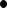	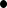	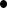	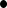	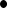		
ZMS	zymosan	TLR2/6, TLR2	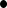	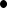	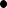	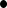			hMonoTLR_LPS

* IRF7 can only be produced after stimulation of combinations of TLR receptors, TLR3 or TLR4 combined with either TLR7, TLR8 or TLR9.

**Figure 1:**
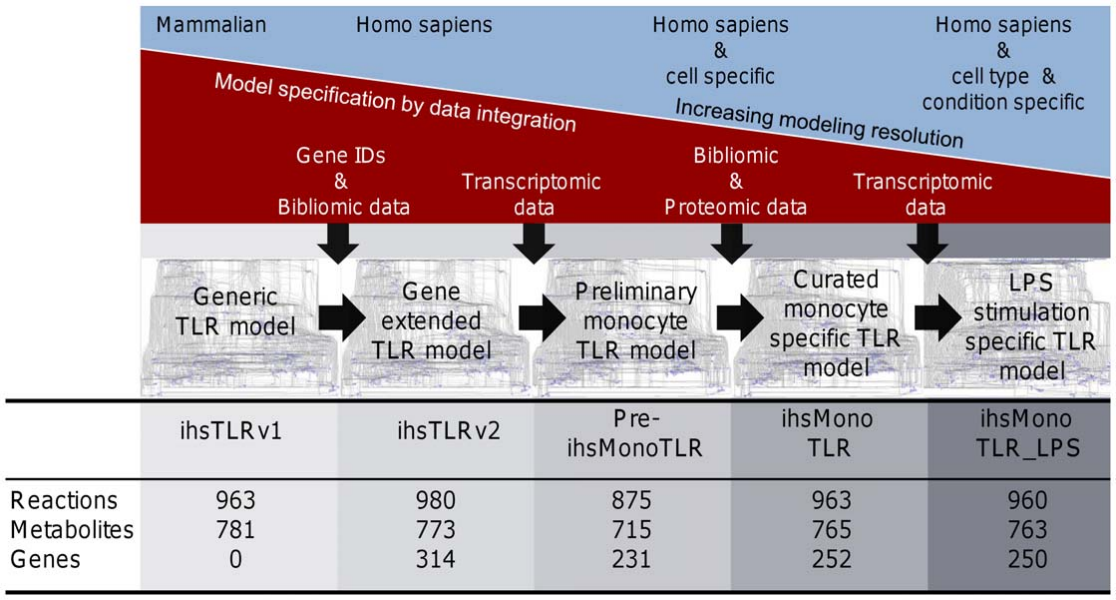
Workflow leading from ihsTLRv1 to a data driven monocyte and LPS stimulated monocyte model. The workflow describes the process of generating cell-type specific, and subsequently cell and condition specific models of TLR signaling in four steps. (1) In the first step, Homo sapiens genes and gene-reaction associations were added to the model. Further, reactions and chemical compounds connected to the signaling of TLR11 were deleted and exchange reactions added. (2) Transcriptomic data was mapped to the model leading to preliminary monocyte specific models of TLR signaling using different cutoffs during the mapping process. (3) The most suitable preliminary model was chosen based on comparison with cell-type specific proteomic data (HPA) and literature evidence. Manual curation was essential to ensure monocyte specific input-output capabilities of the final monocyte model ihsMonoTLR. (4) Transcriptomic data derived from LPS stimulated monocytes was mapped to the ihsMonoTLR to tailor the model condition specific. Statistics on the network sizes at each stage reveal how network size remains comparable while gene contents reduced with increasing modeling resolution.

The aim of the study was to explore the possibility of using COBRA methods and the human TLR signaling network to investigate tissue and disease specific differences in TLR signaling. Therefore, we first identified the set of genes associated with the reactions in ihsTLRv1, and we then generated an updated version of the TLR signaling network (ihsTLRv2). We used expression pattern of the identified TLR genes in human blood derived monocytes to reduce ihsTLRv2 to only contain the cell-type specific set of expressed isoforms, proteins, and reactions (Figure 1, see File S1 for details on the procedure). We then investigate the extent and propagation of the changes induced through LPS stimulation onto pathway utilization.

## Results

### Extensions of gene results in ihsTLRv2

Gene-reaction associations (GRAs), connecting each network reaction with genes encoding participating proteins form the basis for cell-type or condition specific tailoring based on gene expression data. This contextualization was not possible with ihsTLRv1 due to missing GRAs. We employed the NCBI Entrez gene database [27], UniProtKB/Swiss-Prot [28], and primary literature to identify Homo sapiens specific genes and established GRAs using AND and OR Boolean logic. IhsTLRv1 represented mammalian TLR signaling and included TLR1 through TLR11. However, the human open reading frame for TLR11 contains multiple stop codons indicating that this receptor may not be expressed [29]. Therefore, we removed TLR11, ten associated reactions (File S2, Table S1), and eight chemical compounds from the network (File S2, Table S2). We added exchange reactions to resolve dead-ends, i.e., reactants that were only produced or consumed, in the network (File S2, Table S3). Gene extension and tailoring of receptor content led to the human gene extended TLR signaling network, deemed ihsTLRv2. In total, we included 314 genes into ihsTLRv2, of which 312 genes were identified for 178 unique chemical compounds (File S2, Table S4) and two genes associated with a choline uniport reaction were taken from human metabolic reconstruction [30]. The choline uniport transporter encoded by the genes was not a chemical compound in ihsTLRv2. The 178 unique chemical compounds can be divided into receptors (14), kinases (64), phosphatases (7), and the remaining chemical compounds (93), also referred to as other proteins. Receptors were only encoded by single genes, while isoforms were much more common among the kinases (58%), the phosphatases (96%), and the other proteins (63%) (Table 2). Overall, redundant genes comprised 55% of the ihsTLRv2 gene content. We established GRAs for 558 of the 980 ihsTLRv2 reactions. A total of 291 modeling related reactions (i.e., sink, demand, and exchange reactions) were not assigned with GRAs. The remaining reactions without GRAs split into transport reactions of metabolites (37), TLR ligand expression, transport and binding reactions (87), reactions involving generic chemical compounds (3), and orphan chemical compounds (4). Ras family small GTP-binding protein generic (Ras) genes were not included in the current version of ihsTLRv2 due to functional ambiguity. The current version of ihsTLRv2 further did not include gene association for lipopolysaccharide-binding protein (LBP) due to its external origin. The chemical compounds SRC (c-Src), SRCK (Src family kinase (generic)), and SRTK (Src-related tyrosine kinase) were not unambiguously defined in ihsTLRv1. After thorough literature review, we assigned one gene to SRC (c-Src), while we treated SRCK and SRTK as the same chemical compound (File S2, Table S4).

**Table 2:** Statistics of the gene extension of the generic human TLR model.

Groups of Chemical compounds	Chemical compounds (n)	Genes assigned (n)	Unique genes (n)	Redundant genes (n)
Receptors	14	14	14	0
Kinases	64	100	42	58
Phosphatases	7	54	2	52
Proteins	93	144	83	61
Total	178	312	141	171

Redundant genes comprise of all genes, which are associated with chemical compounds having isoforms.

### Generation of a draft monocyte specific TLR model based on gene expression data

**Figure 2:**
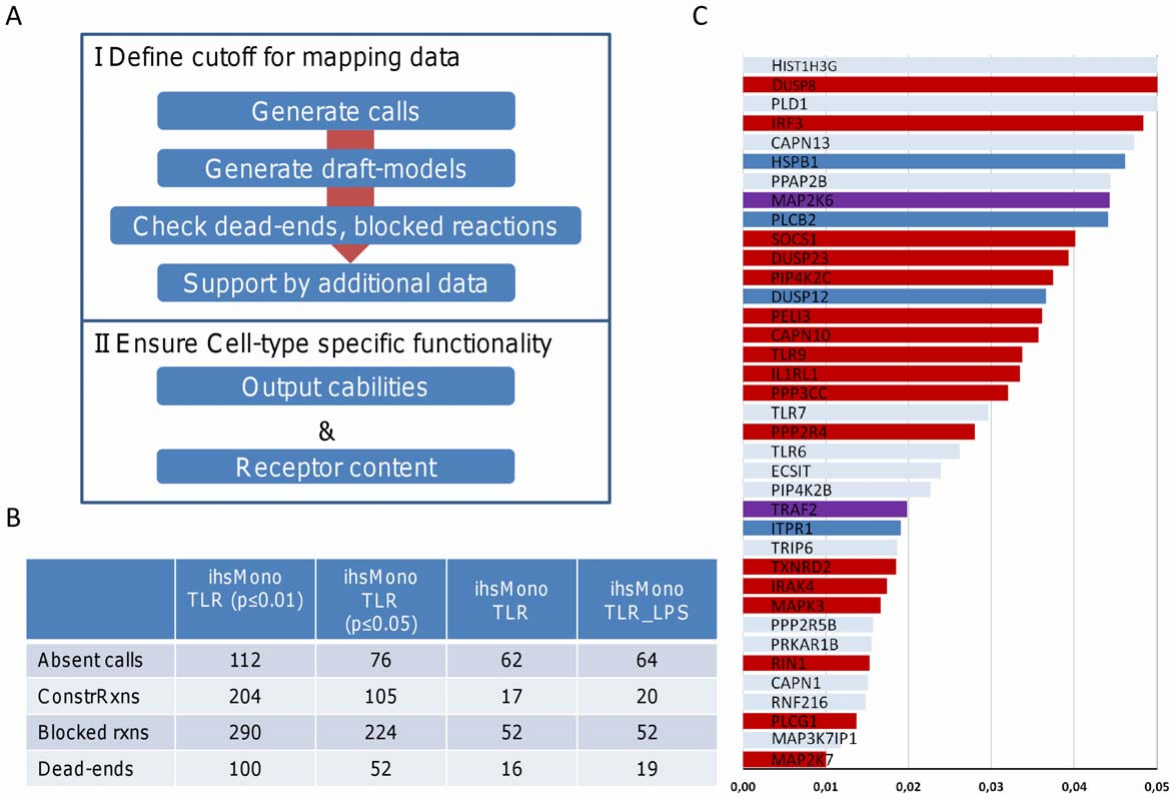
Definition of cutoff for initial monocyte draft-model. **A.** The procedure for the generation of the monocyte specific model was divided into two parts. First, a suitable cutoff was defined for mapping the gene expression data. Therefore, preliminary monocyte models were generated for two cutoffs (p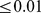 and p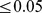). Both cutoffs led to high numbers of blocked reactions and dead-end nodes in the networks. We identified the set of genes only absent in the more stringent cutoff and validated expression of the gene products using the Human Protein Atlas immunohistochemical data of two monocytic leukemia cell lines (THP-1 and U-937) and chose the cutoff, which represented monocyte protein expression the best. The second part of the procedure concerned the assurance of monocyte specific network functionality. Input and output capabilities of the monocyte model were curated according to cell-type specific literature evidence. **B.** Statistic of the number of deleted genes, reactions constrained during the data mapping, blocked reactions, and dead-end nodes in the preliminary monocyte models, the curated monocyte model (ihsMonoTLR), and the LPS stimulation specific monocyte model (ihsMonoTLR_LPS). **C.** Graph illustrating the detection probability of the genes absent in the stringent and present in the moderate cutoff. Genes are colored according to whether they were expressed (red), they were not expressed (blue), no data was available (pale), or data among cell lines was discriminating (purple). In many cases, no data was available, or the proteins were expressed in the cell lines. Only in few cases, the genes were not expressed in any of the cell lines. Also, absent gene expression was distributes across the entire range of the thresholds, such that no intermediate cutoff could be established. As a result, the monocyte model was based on the more moderate cutoff.

In order to derive a monocyte specific model of TLR signaling (ihsMonoTLR), we mapped gene expression data from untreated monocytes [31] onto the network. To find a suitable cutoff, distinguishing between presence and absence of expressed genes, we generated draft-reconstructions based on two different cutoffs p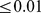 and p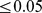. A set of 37 genes solely received absent calls for the more stringent cutoff. The cutoff had a major impact on the number of dead-end metabolites and blocked reactions, i.e., reactions that cannot carry any flux in the network due to dead-end metabolites (Figure 2). The decision for a particular cutoff has therefore a major impact on the network capabilities as well as on the time required for manual curation of the model to ensure similar functionality as in the cell. Protein expression data were obtained for 23 genes in two monocytic leukemia cell lines (THP-1 and U-937) [32]. Most of the genes (17) were moderately expressed, which was mostly the case for both cell lines. The remaining six gene products had not been detected using immunohistochemistry, four of which were absent in both cell lines, while two gene products were only expressed in one of the cell lines. There was no correspondence between statistical detection probability and negative detection that would make us favor the more stringent, p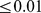, cutoff (Figure 2). Literature search yielded experimental evidence for the presence for four genes (File S2, Table S5). Since the majority of genes rejected by the stringent cutoff was found to be present in monocytes based on immunohistochemistry and literature evidence, we proceeded our network tailoring by using the p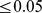 cutoff, as it seemed more suitable for monocytes and the given data set.

### Literature based curation of the draft monocyte specific TLR model

Literature provided evidence for the production of all six outputs in human monocytes [7], [33]–[35]. Using flux variability analysis (FVA) [36], [37] on the draft monocyte TLR model, we found that ROS production was only partly possible through one of two defined output reactions, and that NF-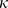
B production was completely impaired (Table 3). We completed the corresponding output pathways by adding the protein kinase C, zeta (EntrezGene ID: 5590), which recovered both outputs (File S2, Table S6) see also Methods section). This gene product is known to be important in NF-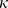
B activation, and its presence in U-937 cells has been demonstrated [38]. Three genes (Entrez Gene ID: 815–818), encoding for isoforms of CaMKII, had direct impact on the models output capabilities. Reincorporation the genes encoding CaMKII resulted in a major increase in CREB output production (File S2, Table S6). We further curated ihsMonoTLR based on known monocyte function, instead of relying solely on a pathway driven approach. Only genes were considered, which were absent in ihsMonoTLR, while isoforms of already captured genes were ignored. Subsequently, 14 genes were reintroduced to the ihsMonoTLR network based on literature support (File S2, Table S7). The final monocyte specific TLR signaling network, ihsMonoTLR, contained 62 genes less than the generic TLR signaling network, ihsTLRv2 (Figure 1). The gene reduction mainly affected the presence and absence of redundant genes, while the signaling pathways mostly remained complete. The genes absent in the final hMonoTLR model encode proteins of 22 chemical compounds in the network (Table 4). We found large decrease in the number of expressed isozymes, e.g., for calpain, which are calcium-dependent cysteine proteases and ubiquitously expressed. Its functions include, among others, pro-IL-1 processing [39]. In total, nine out of the 16 calpain genes were found to not be expressed in the monocytes. Despite the reduced number of genes, functional calpain complexes could still be assembled and the model could produce IL-1.

**Table 3:** Maximum possible flux values for output reactions in the different TLR signaling models.

Output	ihsTLRv2	ihsMonoTLR draft p 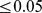	ihsMonoTLR draft p 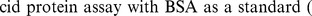	final ihsMonoTLR	ihsMonoTLR_LPS
IRF3	25.00	0.00	0.00	25.00	25.00
IRF7	11.11	11.11	11.11	11.11	11.11
ROS	25.00	25.00	0.00	25.00	25.00
ROS	50.00	50.00	27.00	50.00	50.00
AP-1	25.00	1.00	1.00	25.00	25.00
CRE	12.50	12.50	0.00	12.50	12.50
AP-1	25.00	25.00	0.00	25.00	25.00
NF- 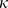 B	14.29	0.00	0.00	14.29	14.29
NF- 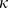 B	14.29	0.00	0.00	14.29	14.29

Fluxes are given in (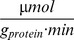).

**Table 4:** Distribution of absent genes.

Chemical compound	Genes absent in hMonoTLR	Genes encoding chemical compound	Group of chemical compound
Ajuba	1	1	Protein
Kinase suppressor of RAS 2	1	1	Kinase
Toll-like receptor 10	1	1	Receptor
Toll-like receptor 3	1	1	Receptor
beta-transducin repeat containing protein 2	1	2	Protein
A20-binding inhibitor of NF- 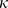 B activation	1	3	Protein
Sarco/endoplasmic reticulum Ca(2+)-ATPase	1	3	Kinase
Serum/glucocorticoid regulated kinase	1	3	Kinase
Thioredoxin reductase	1	3	Protein
Ubiquitin-conjugating enzyme E2D	1	3	Protein
cAMP responsive element binding protein	1	4	Protein
Ubiquitin	1	4	Protein
Protein phosphatase 2B	1	5	Phosphatase
Protein kinase A	1	7	Kinase
Cholin uniport	2	2	Metabolite transporter*
Phosphoinositide 3-kinase	2	6	Kinase
MAP kinase phosphatase	3	16	Phosphatase
Protein Phosphatase 2A	3	17	Phosphatase
Src family kinase/Src-related tyrosine kinase	5	10	Kinase
Ddiacylglycerol kinase (generic)	6	10	Kinase
Histone H3	9	12	Protein
Phosphatidic acid phosphatase (generic)	9	14	Phosphatase
Calpain	9	16	Protein

* Metabolite transporter did not have a chemical compound as the genes were only added to the transport reaction.

During the curation step, the number of dead-end metabolites and blocked reactions was reduced (Figure 2B). The output capabilities of ihsMonoTLR remained equal to ihsTLRv2 (Table 3).

TLR3 and TLR10 were absent in the monocyte specific model, while all other TLRs and NODs were present (Table 1), in agreement with literature [5], [7], [40]–[42].

The wealth of supporting literature evidence for TLR signaling specific components in monocytes underlines that we defined biologically conclusive cutoff for generating a monocyte model of TLR signaling network. We also demonstrated that the monocyte specific model generation required substantial manual curation upon gene expression data mapping to reflect well the known cell-type specific receptor content.

### Tailoring the monocyte TLR model to a LPS stimulation specific model

In monocytes, TLR4 stimulation activates several signaling pathways and transcription factors (TFs) as well as induces inflammatory gene expression programs [33]. In order to investigate this distinct network state, we used gene expression data of the aforementioned experiment [31] to tailor ihsMonoTLR condition specific. Two ihsMonoTLR genes, pellino homolog 3 (PELI3) and TLR6, were no longer expressed upon LPS stimulation. It has been experimentally shown that LPS stimulation led to degradation of the PELI3 gene product in human peripheral blood mononuclear cells and that the protein levels only recovered after several hours [43]. Absence of the PELI3 gene was therefore unlikely to be an artifact. The resulting ihsMonoTLR_LPS model contained 960 reactions and 763 chemical compounds (Figure 1). The number of reactions reduced by three, which were associated with the two absent genes, and two chemical compounds were absent compared to the monocyte model. The number of dead-ends and blocked reactions increased, while functionality with respect to the outputs remained the same (Table 3). Two genes, which were absent in unstimulated monocytes, appeared to be expressed after LPS stimulation. A Src family kinase (Entrez Gene ID: 7525) and TNFAIP3 interacting protein 3 (Entrez Gene ID: 79931). Hence, these genes were not expressed in unstimulated monocytes, they were no longer part of ihsMonoTLR and were not further considered in ihsMonoTLR_LPS. Both of the genes encode isoforms and in both cases at least one other isoform was present in unstimulated and stimulated monocytes. Addition of the genes would therefore not have altered the number of active reactions. This observation highlights that the generation of a truly generic, condition unspecific monocyte model requires a compilation of multiple data sets and that curation of redundant genes would be needed.

### Condition specific network states of monocyte TLR signaling

The monocyte models reconstructed herein allow for simulation and analysis of changes in energy levels and altered gene expression that occur in case of innate immune response. Both cases will be investigated in the following sections.

### Sensitivity analysis

**Figure 3:**
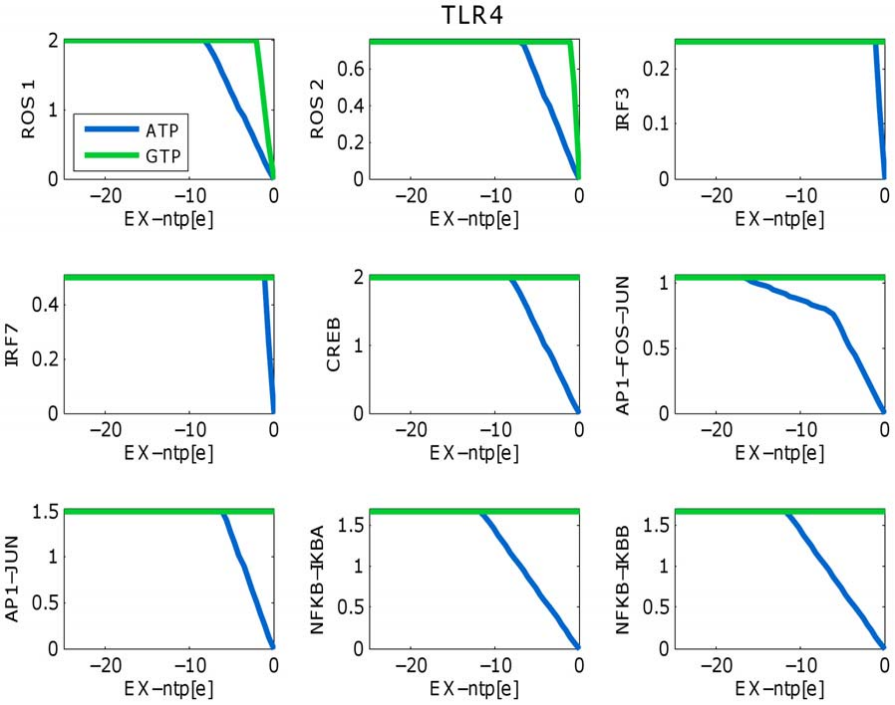
Sensitivity analysis. hMonoTLR_LPS was used for the sensitivity analysis. The network contains nine output reactions for six distinct outputs ROS, IRF3, IRF7, CRE, AP-1, and NF-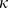
B. ROS,CRE, AP-1, and NF-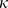
B could be produced by all receptor inputs. Energy dependencies of output production did not differ among input receptors. IRF3 was only produced after stimulation of TLR4. IRF7 was only produced when TLR4 and either TLR7, TLR8 or TLR9 were stimulated together. In case of IRF7, we stimulated the network via TLR4 and TLR8.

Signaling and innate immune response are energy dependent cellular processes. During antibacterial innate immune response, intracellular ATP levels might rapidly deplete [44]. In order to evaluate the energy dependency of the TLR signaling network, we performed a sensitivity analysis testing ATP and guanosinetriphosphate (GTP) requirements of the distinct outputs produced after stimulation through one of 13 input receptors in hMonoTLR_LPS (File S2, Table S8, see also Methods section). We found the same qualitative dependencies on energy species for 12 input receptors and production of ROS, CREB, AP-1 and NF-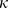
B (File S3). As depicted for TLR4 stimulation (Figure 3), all output production was dependent on ATP, and ROS production further dependent on GTP. TLR4 is the only input receptor in the monocyte model to produce both Interferon regulatory factor 3 (IRF3) and IRF7, which was dependent on ATP but not GTP. NOD receptors produced other outputs beside NF-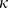
B due to thermodynamically infeasible loops in the network (discussed in [24]) which are necessary for network function ( File S3, Figures S2, S3). Production of NF-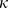
B from NOD receptor (NOD1 and NOD2) stimulation was ATP- but not GTP-dependent. This sensitivity analysis demonstrates the requirement of the TLR model for ATP and GTP and that the availability of energy could indeed modulate the signaling outputs.

### Setting quantitative gene expression changes into context

Quantitative changes in gene expression could possibly alter flux distributions and produced outputs within the network. Compared to the relatively small differences in qualitative gene expression, LPS stimulation induced up-regulation of 28 ihsMonoTLR_LPS genes (File S2, Table S9) and down-regulation of three genes (File S2, Table S10). Together, they represented 12% of the genes. Of the 28 up-regulated genes, ten encoded isoforms. None of the down-regulated genes were isoforms. We called a gene differentially expressed when at least 50% of the probe sets of the gene were differentially expressed. Eight genes, three up- and five down-regulated with regulated probe sets, were rejected due to this threshold and will be referred to as subthreshold genes in the following sections. Subthreshold genes represented further 3% of the ihsMonoTLR_LPS genes. Taken together, only a small number of the ihsMonoTLR_LPS genes showed altered gene expression level two hours post-stimulation with LPS.

#### Estimation of the impact of the up-regulated genes on network topology

**Figure 4:**
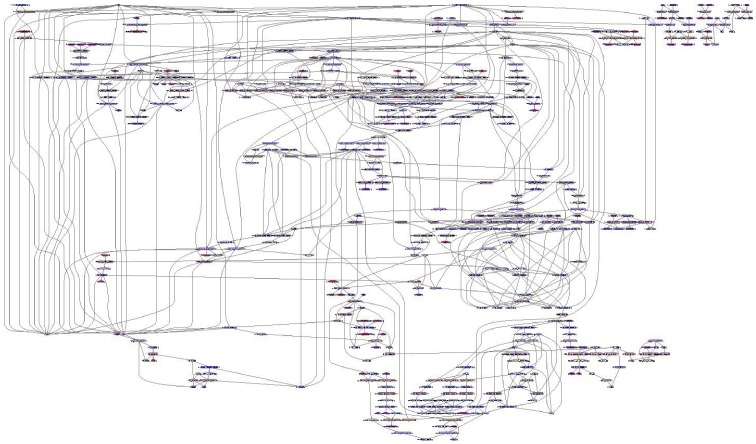
Network resulting from mapping of the up-regulated genes onto the LPS stimulation specific monocyte model. We extracted a sub-network from LPS stimulation specific monocyte model (ihsMonoTLR_LPS) consisting of all reactions associated with the 28 up-regulated genes, which included 19% of the reactions and 39% of the chemical compounds of ihsMonoTLR_LPS. The visualization revealed a comprehensively connected network. Details can be viewed using the file provided in the supporting information (File S4). Network illustration was generated using software Paint4Net [65].

**Figure 5:**
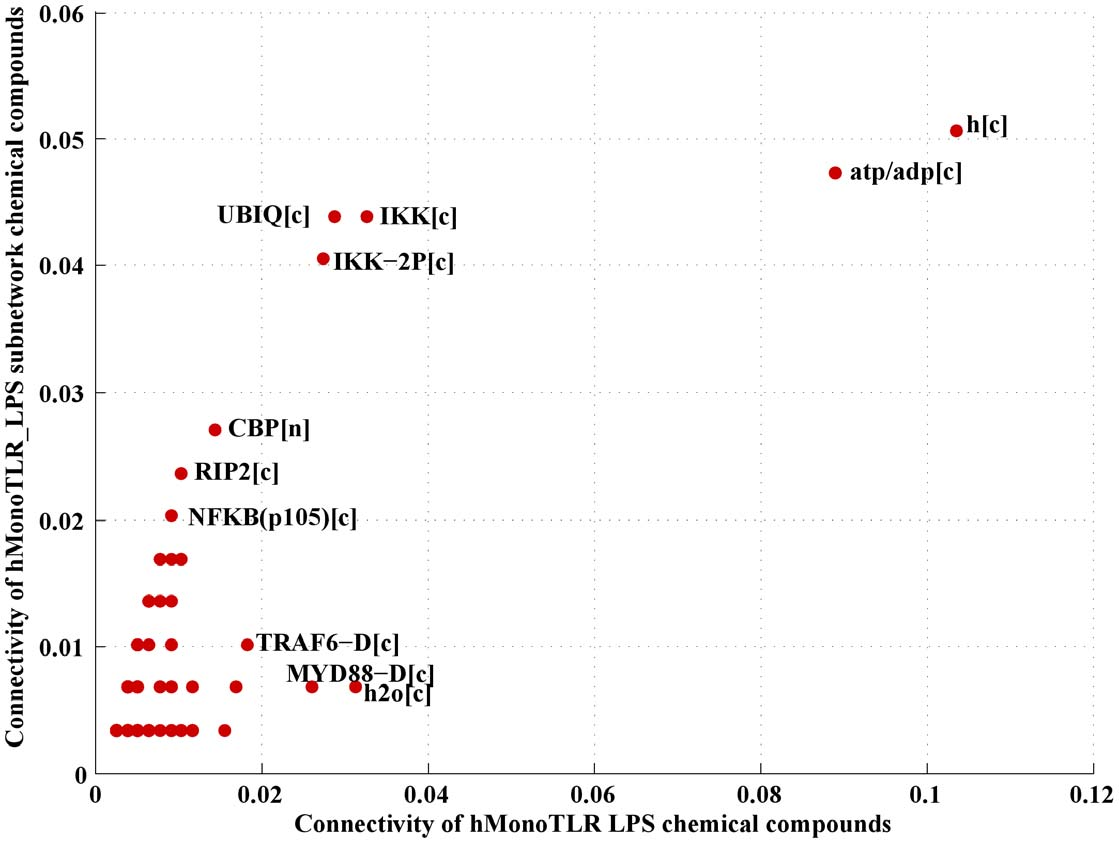
Comparison of (chemical compound) connectivity in the LPS stimulation specific versus the up-regulated sub-network. We report the connectivity as a ratio of compound 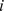 and 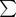
(chemical compounds) in the respective model (ihsMonoTLR_LPS subnetwork and ihsMonoTLR_LPS).

We were interested in the impact of the regulated genes on the TLR signaling network functionality. Since ihsMonoTLR_LPS represents accurately the functions of each gene product, we extracted a sub-network consisting of all reactions associated with the 28 up-regulated genes (ihsMonoTLR_LPS_upreg), which included 185 reactions (19% of ihsMonoTLR_LPS) and 296 chemical compounds (39% of ihsMonoTLR_LPS) (Figure 4). The sub-network also included output reactions for NF-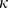
B and AP-1 implying an influence of the up-regulated gene set, in particular, upon these two different model outputs, which is in agreement with experimental data [33], [45]. We compared the connectivity of the chemical compounds within the sub-network with ihsMonoTLR_LPS. The high metabolite connectivity of protons, ATP, and ADP was conserved in the sub-network, even though the relative connectivity was smaller in the sub-network than in ihsMonoTLR_LPS (Figure 5). Chemical compounds, such as ubiquitin and the inhibitor of the kappa light polypeptide gene enhancer in B-cells kinase (IKK), had lower numbers of connections compared to ihsMonoTLR_LPS, but in relation to the number of chemical compounds in ihsMonoTLR_LPS (n = 763) and the sub-network (n = 296) relative connectivity was higher in the sub-network. In contrast, we found chemical compounds, such as TRAF-6 and MyD88, to be higher connected in ihsMonoTLR_LPS. These differences in the connectivity arose since the set of up-regulated genes centered on NF-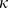
B activation, while the chemical compounds with higher relative connectivity in the ihsMonoTLR_LPS appear more up-stream in the signaling cascades of the network. Since the ihsMonoTLR_LPS_upreg comprises of all reactions and functions that are higher used upon LPS stimulation, they can be interpreted as the active sub-network used by the monocytes to process the information and initiate the corresponding program. The high connectivity in ihsMonoTLR_LPS_upreg indicates that the retrieved sub-network mediates NF-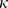
B activation subsequent to LPS stimulation.

#### Analysis of the down-regulated sub-network module

**Figure 6:**
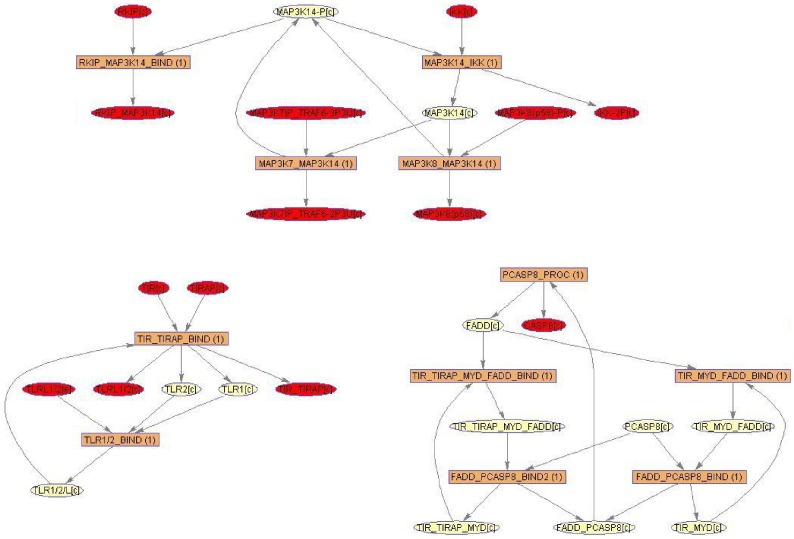
Network modules resulting from mapping of the down-regulated genes onto the LPS stimulation specific monocyte TLR model. The sub-network that was extracted from ihsMonoTLR_LPS based on the three down-regulated genes comprised of 26 metabolites and eleven reactions. Illustration of the sub-network revealed three separated modules confirming that the impact of down-regulation, based on involvement, was rather small. Network illustration was generated using software Paint4Net [65].

Sub-network extraction was also performed based on the three down-regulated genes. The resulting sub-network comprised eleven reactions and 26 chemical compounds (Figure 6). It did not include any output reaction. The impact of down-regulation, based on involvement, as we assessed in the previous section, was rather small. The sub-network consisted of three separated modules centering either mitogen-activated protein kinase kinase kinase 14 (MAP3K14), TLR1, or Fas (TNFRSF6)-associated via death domain (FADD). In case of FADD, another gene product of a minority gene and not used for sub-network extraction, appeared in this context as a direct interaction partner, i.e., caspase-8 (CASP8). CASP8 is known to interact with FADD in monocytes, as part of the differentiation pathway, and to prevent sustained NF-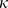
B activation along the macrophage differentiation [46]. This example shows how ihsMonoTLR can serve as a resource for context-specific analysis by providing functional relationships.

### Functional representation of quantitative changes induced through LPS stimulation

We used the computed fold changes (FCs) to represent the LPS activated state of ihsMonoTLR_LPS. Up- and down-regulation was mimicked by either enforcing the minimal reaction flux or reducing the possible maximum flux through reactions associated with regulated genes. Mapping was performed separately for each of 117 I/O relationships covering 13 input reactions and 9 output reactions in hMonoTLR_LPS (File S2, Table S8, see also Methods section). Subsequently we assessed the consequences of gene regulation based on the altered flux ranges of the 9 output reactions, obtained through FVA. The TLR model contains thermodynamically infeasible loops [24], which cause baseline flux through output reactions in the model. First, we investigated the effect of stimulation beyond baseline flux values for each I/O relationship. Therefore, we subtracted fluxes derived after stimulation from the baseline fluxes (File S2, Table S11). The pattern of outputs produced by stimulation of an input was, as expected, in the majority of cases. Stimulation caused flux through ROS, CREB, AP-1, and NF-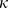
B for all TLRs and for IL1R1. Stimulation of TLR4 additionally induced IRF3. Combined stimulation of TLR4 and TLR8 led to IRF7. Stimulation of NOD receptors produced NF-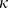
B and AP-1 could be produced (through ‘AP1_FOS_JUN_BIND’) after stimulation of NOD receptors. After we confirmed the I/O relationships, we went on to investigate the effect of quantitative gene expression changes onto output production. In total, 183 reactions were associated with regulated genes, whereof only a subset was active in a particular I/O relationship. As expected, mapping of differential expression onto the network enforced AP-1 and NF-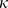
B production across all ihsMonoTLR_LPS inputs, as genes directly associated with the output reactions of AP-1 and NF-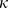
B were up-regulated (File S2, Table S12). Flux was further enforced through AP-1 output reactions (‘AP1_FOS_JUN_BIND’, and ‘AP1_JUN_BIND’) equally for all 13 inputs, except for NOD receptors. We predicted a lower flux through ‘AP1_FOS_JUN_BIND’ when the NOD receptors were stimulated than for the other receptors. Data mapping enforced the production of IRF3 output in the model after TLR4 stimulation, and IRF7 after stimulation through TLR4 and TLR8. ROS and CREB output production was not affected by the mapping of differentially expressed genes. Among the output reactions, no effects of the mapping of down-regulation were observed. This analysis demonstrated how the model can be used to predict differences in cellular phenotypes due to quantitative gene expression differences.

## Discussion

The aim of this study was to establish a method for omics data driven contextualization of signaling networks after gene-extension of the human TLR signaling network (Figure 1, see File S1 for details on the procedure). Our key results demonstrate that i) substantial manual curation is required after specializing the generic TLR signaling network to a cell-type and condition specific sub-network; ii) the monocyte TLR signaling network captured most of the functionality of the generic network but gene redundancy was removed, indicating cell-type specific use of isoforms; and iii) TLR signaling is highly energy dependent as all TLR signaling pathways required ATP availability and ROS production was additionally dependent on GTP availability. Taken together, we demonstrated that the contextualization of the TLR network enables the functional analysis of TLR signaling in health and disease.

We employed the gene-extended TLR signaling network together with gene expression data and literature evidence, which ensured monocyte specific functionality with respect to I/O pathway content (Table 1). The role of manual curation work has been emphasized as important step in the generation of a biological meaningful cell-type or tissue specific models, despite a growing number of sophisticated algorithms [47]. Curation with respect to the function was important as the monocyte model was the template for subsequent, condition specific tailoring and analysis of the consequences of LPS stimulation for network structure and function. The TLR expression in monocytes at the chosen cutoff and cell-type specific literature were found to be in good agreement with some but not all experimental studies [5], [7] indicating the importance of reproducible, consistent experimental conditions and of using identical monocyte subsets. For instance, infection states or stimulation can drastically alter cellular processes and induce the production of effector molecules, such as cytokines [9], for which the cell has to provide energy for the transcriptional and translational machinery. Such cellular changes can even involve usage of central metabolism pathways, including the switch to glycolysis for faster energy allocation [48], [49]. IhsTLRv2 was redundant in its pathways connecting inputs with specific sets of outputs and with respect to genes encoding isoforms. Transition from ihsTLRv2 to cell-type specific ihsMonoTLR was characterized by isoform reduction, while network size remained comparable. This may be partly due to not manually curating the expression state of isoforms. Monocytes describe cells that are central to the host innate immune defense and are known to express many TLRs [4], [10], [50]. Our finding that the majority of the signaling network is preserved in monocytes is thus plausible. Transition from unstimulated to the LPS stimulated model of TLR signaling was characterized by only few qualitative differences but prevailing differences in quantitative gene expression was observed. The set of up-regulated genes was found to be tightly connected (Figure 4). The impact of the up-regulated genes spread across one third of ihsMonoTLR_LPS depicting the strong influence that LPS stimulation has upon the monocyte TLR signaling network. The LPS stimulation specific sub-network of up-regulated genes correctly contained the transcription factors NF-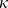
B and AP-1 as their activation is an expected response of a monocyte to LPS stimulation [33], [51].

TLR signaling is highly energy dependent as demonstrated with the sensitivity analysis (Figure 3). TLR signaling network accounts for a number of other metabolites that link it to further metabolic processes. Integrating of models of different cellular processes, such as metabolism, signaling, and gene regulatory networks [52]–[55], will enable important insights into the crosstalk between signaling and metabolism. Corresponding modeling tools are currently developed [18], [56], [57]. In fact, the interaction between metabolism and innate immunity is of great interest both for health and disease [49]. For instance, TLR agonists can stimulate a switch from oxidative phosphorylation to glycolysis in murine dendritic cells and macrophages [48], [49]. This switch lead to faster yet less effective ATP production, similar to the Warburg effect observed in cancer cells, and may function as a protective mechanism to preserve cellular ATP levels and maintain cell viability and function during an immune response [48], [49]. Moreover, it has been suggested that neuronal TLR signaling is involved in triggering cell death in response to brain injury [6]. Combined signaling and metabolic COBRA modeling could help consolidating the complexity of the diseases by highlighting cross-relations.

Taken together, we demonstrated that a stoichiometric model of the TLR signaling network combined with transcriptomic data can provide functional insight into its signaling cascades. The presented gene extension and method to integrate transcriptomic data opens up an alley for more detailed, disease directed research, including drug target discovery, and thus rendering signaling models amenable to similar contextualization as already established for metabolic models.

## Materials and Methods

### Gene extension

Genes for chemical components were identified using NCBI Entrez gene database [27], UniProtKB/Swiss-Prot [28], and primary literature. The generation of Gene-Reaction Associations (GRAs) was subsequently performed using the rBioNet software [58]. The rBioNet software requires a gene index file. The gene index file contains Entrez gene ID, gene Symbol, location, gene type and description of added model genes and of the genes encoding the members of the Ras family. To generate the gene index file (File S2, Table S13), Homo sapiens gene information was downloaded from NCBI (4/13/2011). The software allows loading model structures and to easily alter the model content, such as reactions and GRAs. Genes were associated with reactions using Boolean logic, AND for complexes requiring multiple subunits and reactions requiring multiple proteins. OR was assigned for functional isoforms.

### Gene association additional information

Ras protein family is encoded by 35 genes [59], but they were not included in the current version of ihsTLRv2 due to functional ambiguity. However, the genes were included into the gene index file (File S2, Table S13), and can easily be added rBioNet [58]. Additionally, no gene associations were added for reactions involving lipopolysaccharide-binding protein (LBP), which has been described as protein produced in the liver and transported in the blood [60], [61]. Cytokine production can even be induced in absence of LBP, as was demonstrated for monocytes stimulated with LPS in presence of rsCD14 [62]. The primary purpose here was to enable data mapping, and by not adding the gene we ensured that absence of the LBP gene in gene expression data could not interfere with TLR4 signaling. The LBP gene was included in the gene index file so it could easily be added. Due to the lack of gene reaction association, reactions connected to these chemical compounds will be always active when data is mapped onto the network.

### Model tailoring

In addition to the identification and association of model reactions with human genes, a number of reactions were removed from the model content using rBioNet software [58] in order to tailor the model human specific. The removed reactions concerned the transmission of input signal from TLR11 stimulation. Furthermore, 27 exchange reactions were added for dead-end chemical compounds such as extracellular invaders, IL1A and IL1B (File S2, Table S3).

### Analysis of gene expression data and mapping

Gene expression data for unstimulated and LPS stimulated human monocytes were obtained from Gene Expression Omnibus (http://www.ncbi.nlm.nih.gov/geo/). We employed data from two experimental groups (vehicle (control), LPS low 2 hrs) [31]. Three chips were excluded from the analysis (GSM252451, GSM252454, and GSM252479) after visual inspection. Absence and presence calls, dividing the set of ihsTLRv2 genes into sets of expressed (present) and unexpressed (absent) genes were generated using the PANP package [63], R (2.13.0) computational platform [64], and using Affymetrix annotation files, for the loose cutoff p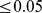 and for the stringent cutoff p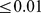. For genes with multiple identifiers, we only used the identifier showing the highest mean expression intensity in the control group (File S2, Table S14). Therefore, it is more likely to assign presence calls to absent genes than the other way around. Lists of absent genes used for generation of the preliminary and final monocyte (hMonoTLR) and LPS_stimulated monocyte model (hMonoTLR_LPS) are provided in the supplementary information (File S2, Table S15). For the mapping of the transcriptomic data, we took advantage of the previously defined GRAs. Reactions were disabled that were associated with gene products that had an absence call associated. In case of functional isoforms, reactions were only disabled if all isoforms were called absent. This way the protein and reaction content of the TLR network was reduced to form the preliminary monocyte models of two different cutoffs.

#### Cutoff-definition

The Human Protein Atlas (http://www.proteinatlas.org/) was queried using gene symbols of 33 genes with different P/A calls using two different gene expression cutoffs (File S2, Table S15), for expression of the encoded proteins in two monocytic leukemia cell lines, THP-1 and U-937. If the corresponding antibody yielded at least weakly staining for the majority of tests in one cell sample, we called the protein present.

#### I/O pathway curation using illustration tool

In order to enable all I/O pathways in the monocyte draft-model, network reactions connecting missing outputs to input were identified, using software Paint4Net [65]. This tool facilitated curation of incomplete I/O relationships in ihsMonoTLR. We first derived a list of reactions involved in the signaling pathway towards NF-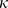
B using ihsTLRv2, which contained the complete pathways, as reference. Subsequently, we did the same for the uncurated ihsMonoTLR and the disconnected output pathways. Comparison of the resulting list of participating reactions revealed the missing links in ihsMonoTLR. Through the GPAs of missing reactions we quickly identified six candidate genes with potential impact on output production. Reincorporation of a single gene at a time revealed the impact of the absence of the particular gene on the output capability of the model.

#### Sensitivity analysis

All exchange reactions of ligands and Ligand to receptor binding reactions (File S2, Table S8) in hMonoTLR_LPS were constraint to zero 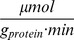. To simulate the distinct I/O relationships, input combinations were as follows, ‘EX_26dap-LL[e] and ‘NOD1P_BIND’, ‘EX_ALPS[e]’ and ‘TLR2/L-D_BIND’, ‘EX_LPS_HS[e]’ and ‘TLR4/L_MD2_BIND’, ‘EX_FLGN[e]’ and ‘TLR5_BIND’, ‘EX_TCLDLPP[e]’ and ‘TLR1/2_BIND’, ‘EX_BPM[e]’ and ‘TLR7_BIND’, ‘EX_SSRNA[e]’ and ‘TLR8_BIND’, ‘EX_UMLCPGD[e]’ and ‘TLR9_BIND’ or ‘TLR9_BINDII’, ‘EX_MRDP[e]’ and ‘NOD2P_BIND’). For interleukin-1, no exchange existed to specifically drive IL1R1 stimulation. We therefore added an exchange reaction for IL1R1 (‘EX_IL1R1_LIG[e]’). This exchange reaction was enabled in combination with ‘IL1R1_BIND’ in order to simulate single receptor IL1R1 stimulation. The nine output reactions were ‘DM_PHOX_GTP-3P[v]’, ‘DM_PHOX_GTP-8P[v]’, ‘DM_ISRE_IRF3[n]’, ‘DM_ISRE_IRF7[n]’, ‘CREB_CRE_BIND’, ‘AP1_FOS_JUN_BIND’, ‘AP1_JUN_BIND’, ‘NFKB_IKBA_DISS’, and ‘NFKB_IKBB_DISS’. As implemented in the network structure of the TLR model, IRF7 output could only carry flux if at least two different inputs were activated ((TLR4) and (TLR7 or TLR8 or TLR9 or TLR9II)). Thus, in case of IRF7, we additionally enabled flux through ‘TLR8_BIND’ and ‘EX_SSRNA[e]’. Note that flux through the remaining output reactions remained possible. To simulate the energy requirements of the I/O relationships, we enabled one exchange reaction (lb = ub = −1 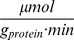) and one corresponding binding reaction (lb = ub = 1 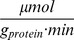) of the specified input combinations and used the COBRA robustness analysis function. Either atp or gtp exchange reaction was the reaction of interest, and nPoints = 50. Sensitivity analysis was performed for each I/O relationship. Prior to analysis atp and gtp exchange reactions were constraint to lb = −25 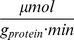 and ub = 0 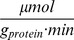.

#### Quantitative gene expression analysis

Gene expression data for unstimulated and LPS stimulated human monocytes were obtained from Gene Expression Omnibus (http://www.ncbi.nlm.nih.gov/geo/). We employed data from two experimental groups (vehicle (control), LPS low 2 hrs) [31]. Three chips were excluded from the analysis (GSM252451, GSM252454, and GSM252479) after visual inspection. Lists of up-and down-regulated genes (File S2, Table S9, S10) were generated using twofold change and p
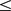
0.05 FDR for min 50% of the identifiers per gene cutoffs using AltAnalyze_v2.02beta for processing of the data [66] using default settings, EnsMart65 database and affymetrix annotation files.

#### Mapping of quantitative expression changes

For this analysis, exchanges were closed and the same I/O relationships used as described for the sensitivity analysis. However, for this analysis, Energy supply of the model was restricted to exchange of atp ub = 0 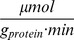 , lb = −100 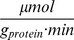) and exchange of gtp ub = 0 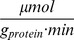, lb = −50 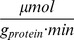). For each I/O relationship, FBA was run using the minNorm option. The sets of model reactions connected to the up-regulated and down-regulated genes were identified and assigned with a fold change (FCrnx). FCrxn was derived from the change in expression of the regulated gene that was associated with a reaction. If more than one gene associated with a reaction was significantly regulated, the mean fold change was calculated. Highest fold change for up-and down-regulation in the data set served as a reference fold change (FC-up and FC-down). Reaction bounds were adjusted based on the following equations (1) model.lb = 
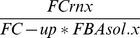
 and (2) model.ub = 
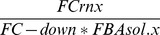
. We compared minimum and maximum flux values derived through FVA [37] for each of the nine output reactions in response to stimulation through each of the input receptor types.

All computations were carried out using Matlab (Mathworks, Inc), the COBRA toolbox [14], and TomOpt (Tomlab, Inc) as linear programming solver.

## Supporting Information

File S1 **Workflow describing the generation of a cell-type specific network of TLR signaling in a step by step manner.**
(PDF)

File S2 **Tables (S1–15) of supporting information used at each step of gene extension, model tailoring and simulations.**
(XLSX)

File S3 **Sensitivity analysis figures for all input/output relationships (Figures S1, S2, S3, S4, S5, S6, S7, S8, S9, S10, S11, S12, S13).**
(PDF)

File S4 **Source figure file of ihsMonoTLRsub_up. It contains the detailed map of the ihsMonoTLRsub_up sub-network.**
(FIG)

## References

[1] Kawai T, Akira S (2006) TLR signaling. Cell Death & Differentiation 13: 816–825. 16410796 10.1038/sj.cdd.4401850

[2] Ospelt C, Gay S (2010) TLRs and chronic inammation. The International Journal of Biochemistry & Cell Biology 42: 495–505. 19840864 10.1016/j.biocel.2009.10.010

[3] Rakoff-Nahoum S, Medzhitov R (2008) Toll-like receptors and cancer. Nature Reviews Cancer 9: 57–63. 19052556 10.1038/nrc2541

[4] Zarember K, Godowski P (2002) Tissue expression of human Toll-like receptors and differential regulation of Toll-like receptor mRNAs in leukocytes in response to microbes, their products, and cytokines. The Journal of Immunology 168: 554–561. 11777946 10.4049/jimmunol.168.2.554

[5] Kadowaki N, Ho S, Antonenko S, de Waal Malefyt R, Kastelein R, et al (2001) Subsets of human dendritic cell precursors express different Toll-like receptors and respond to different microbial antigens. The Journal of Experimental Medicine 194: 863–869. 11561001 10.1084/jem.194.6.863 PMC2195968

[6] Tang S, Arumugam T, Xu X, Cheng A, Mughal M, et al (2007) Pivotal role for neuronal Toll-like receptors in ischemic brain injury and functional deficits. Proceedings of the National Academy of Sciences 104: 13798–13803. 10.1073/pnas.0702553104 PMC1959462 17693552

[7] Cros J, Cagnard N, Woollard K, Patey N, Zhang S, et al (2010) Human CD14dim monocytes patrol and sense nucleic acids and viruses via TLR7 and TLR8 receptors. Immunity 33: 375–386. 20832340 10.1016/j.immuni.2010.08.012 PMC3063338

[8] Dunne A, Marshall N, Mills K (2011) TLR based therapeutics. Current Opinion in Pharmacology 11: 404–411. 21501972 10.1016/j.coph.2011.03.004

[9] Serbina N, Jia T, Hohl T, Pamer E (2008) Monocyte-mediated defense against microbial pathogens. Annual Review of Immunology 26: 421–452. 10.1146/annurev.immunol.26.021607.090326 PMC2921669 18303997

[10] Auffray C, Sieweke M, Geissmann F (2009) Blood monocytes: development, heterogeneity, and relationship with dendritic cells. Annual Review of Immunology 27: 669–692. 10.1146/annurev.immunol.021908.132557 19132917

[11] Dinarello C (2011) Interleukin-1 in the pathogenesis and treatment of inammatory diseases. Blood 117: 3720–3732. 21304099 10.1182/blood-2010-07-273417 PMC3083294

[12] Palsson B (2006) Systems biology : properties of reconstructed networks. Cambridge ; New York: Cambridge University Press, xii, 322 p.

[13] Thiele I, Palsson B (2010) A protocol for generating a high-quality genome-scale metabolic reconstruction. Nature Protocols 5: 93–121. 20057383 10.1038/nprot.2009.203 PMC3125167

[14] Schellenberger J, Que R, Fleming R, Thiele I, Orth J, et al (2011) Quantitative prediction of cellular metabolism with constraint-based models: the COBRA Toolbox v2.0. Nature Protocols 6: 1290–1307. 21886097 10.1038/nprot.2011.308 PMC3319681

[15] Shlomi T, Cabili M, Herrgard M, Palsson B, Ruppin E (2008) Network-based prediction of human tissue-specific metabolism. Nature Biotechnology 26: 1003–1010. 10.1038/nbt.1487 18711341

[16] Becker S, Palsson B (2008) Context-specific metabolic networks are consistent with experiments. PLoS Computational Biology 4: e1000082. 18483554 10.1371/journal.pcbi.1000082 PMC2366062

[17] Jensen P, Papin J (2011) Functional integration of a metabolic network model and expression data without arbitrary thresholding. Bioinformatics 27: 541–547. 21172910 10.1093/bioinformatics/btq702 PMC6276961

[18] Thiele I, Jamshidi N, Fleming R, Palsson B (2009) Genome-scale reconstruction of Escherichia coli's transcriptional and translational machinery: a knowledge base, its mathematical formulation, and its functional characterization. PLoS Computational Biology 5: e1000312. 19282977 10.1371/journal.pcbi.1000312 PMC2648898

[19] Thiele I, Fleming R, Bordbar A, Schellenberger J, Palsson B (2010) Functional characterization of alternate optimal solutions of Escherichia coli's transcriptional and translational machinery. Biophysical Journal 98: 2072–2081. 20483314 10.1016/j.bpj.2010.01.060 PMC2872367

[20] Gianchandani E, Papin J, Price N, Joyce A, Palsson B (2006) Matrix formalism to describe functional states of transcriptional regulatory systems. PLoS Computational Biology 2: e101. 16895435 10.1371/journal.pcbi.0020101 PMC1534074

[21] Gianchandani E, Joyce A, Palsson B, Papin J (2009) Functional States of the genome-scale Escherichia coli transcriptional regulatory system. PLoS Computational Biology 5: e1000403. 19503608 10.1371/journal.pcbi.1000403 PMC2685017

[22] Papin J, Palsson B (2004) The JAK-STAT signaling network in the human B-cell: an extreme signaling pathway analysis. Biophysical Journal 87: 37–46. 15240442 10.1529/biophysj.103.029884 PMC1304358

[23] Dasika M, Burgard A, Maranas C (2006) A computational framework for the topological analysis and targeted disruption of signal transduction networks. Biophysical Journal 91: 382–398. 16617070 10.1529/biophysj.105.069724 PMC1479062

[24] Li F, Thiele I, Jamshidi N, Palsson B (2009) Identification of potential pathway mediation targets in Toll-like receptor signaling. PLoS Computational Biology 5: e1000292. 19229310 10.1371/journal.pcbi.1000292 PMC2634968

[25] Richard G, Belta C, Julius A, Amar S (2012) Controlling the Outcome of the Toll-Like Receptor Signaling Pathways. PLoS ONE 7: e31341. 22363624 10.1371/journal.pone.0031341 PMC3282698

[26] Bajikar SS, Janes KA (2012) Multiscale Models of Cell Signaling. Annals of Biomedical Engineering 10.1007/s10439-012-0560-1 PMC3436998 22476894

[27] Maglott D, Ostell J, Pruitt KD, Tatusova T (2011) Entrez gene: gene-centered information at NCBI. Nucleic Acids Research 39: D52–D57. 21115458 10.1093/nar/gkq1237 PMC3013746

[28] Apweiler R, Martin MJ, O'Donovan C, Magrane M, Alam-Faruque Y, et al (2011) Ongoing and future developments at the Universal Protein Resource. Nucleic Acids Research 39: D214–D219. 21051339 10.1093/nar/gkq1020 PMC3013648

[29] Zhang D, Zhang G, Hayden M, Greenblatt M, Bussey C, et al (2004) A Toll-like receptor that prevents infection by uropathogenic bacteria. Science 303: 1522–1526. 15001781 10.1126/science.1094351

[30] Duarte N, Becker S, Jamshidi N, Thiele I, Mo M, et al (2007) Global reconstruction of the human metabolic network based on genomic and bibliomic data. Proceedings of the National Academy of Sciences 104: 1777–1782. 10.1073/pnas.0610772104 PMC1794290 17267599

[31] Dower K, Ellis D, Saraf K, Jelinsky S, Lin L (2008) Innate immune responses to TREM-1 activation: overlap, divergence, and positive and negative cross-talk with bacterial lipopolysaccharide. The Journal of Immunology 180: 3520–3534. 18292579 10.4049/jimmunol.180.5.3520

[32] Berglund L, Björling E, Oksvold P, Fagerberg L, Asplund A, et al (2008) A genecentric Human Protein Atlas for expression profiles based on antibodies. Molecular & Cellular Proteomics 7: 2019–27. 18669619 10.1074/mcp.R800013-MCP200

[33] Guha M, Mackman N (2001) LPS induction of gene expression in human monocytes. Cellular Signalling 13: 85–94. 11257452 10.1016/s0898-6568(00)00149-2

[34] Izaguirre A, Barnes B, Amrute S, Yeow W, Megjugorac N, et al (2003) Comparative analysis of IRF and IFN-alpha expression in human plasmacytoid and monocyte-derived dendritic cells. Journal of Leukocyte Biology 74: 1125–1138. 12960254 10.1189/jlb.0603255

[35] Cachia O, Benna J, Pedruzzi E, Descomps B, Gougerot-Pocidalo M, et al (1998) *α*-tocopherol inhibits the respiratory burst in human monocytes. Journal of Biological Chemistry 273: 32801–32805. 9830025 10.1074/jbc.273.49.32801

[36] Orth J, Thiele I, Palsson B (2010) What is ux balance analysis? Nature Biotechnology 28: 245–248. 10.1038/nbt.1614 PMC3108565 20212490

[37] Gudmundsson S, Thiele I (2010) Computationally efficient ux variability analysis. BMC Bioinformatics 11: 489. 20920235 10.1186/1471-2105-11-489 PMC2963619

[38] Rahman M, McFadden G (2006) Modulation of tumor necrosis factor by microbial pathogens. PLoS Pathogens 2: e4. 16518473 10.1371/journal.ppat.0020004 PMC1383482

[39] Kavita U, Mizel S (1995) Differential sensitivity of interleukin-1*α* and-*β* precursor proteins to cleavage by calpain, a calcium-dependent protease. Journal of Biological Chemistry 270: 27758–27765. 7499244 10.1074/jbc.270.46.27758

[40] Farina C, Theil D, Semlinger B, Hohlfeld R, Meinl E (2004) Distinct responses of monocytes to Toll-like receptor ligands and inammatory cytokines. International immunology 16: 799–809. 15096475 10.1093/intimm/dxh083

[41] Lech M, Avila-Ferrufino A, Skuginna V, Susanti H, Anders H (2010) Quantitative expression of RIG–like helicase, NOD-like receptor and inammasome-related mRNAs in humans and mice. International Immunology 22: 717–728. 20584763 10.1093/intimm/dxq058

[42] Ogura Y, Inohara N, Benito A, Chen F, Yamaoka S, et al (2001) NOD2, a NOD1/Apaf-1 family member that is restricted to monocytes and activates NF-*κ*B. Journal of Biological Chemistry 276: 4812–4818. 11087742 10.1074/jbc.M008072200

[43] Moynagh P (2009) The Pellino family: IRAK E3 ligases with emerging roles in innate immune signalling. Trends in immunology 30: 33–42. 19022706 10.1016/j.it.2008.10.001

[44] Cohn Z, Benson B (1965) The differentiation of mononuclear phagocytes Morphology, cytochemistry, and biochemistry. The Journal of experimental medicine 121: 153–170. 14253481 10.1084/jem.121.1.153 PMC2137972

[45] Krappmann D, Wegener E, Sunami Y, Esen M, Thiel A, et al (2004) The I*κ*B kinase complex and NF-*κ*B act as master regulators of lipopolysaccharide-induced gene expression and control subordinate activation of AP-1. Molecular and Cellular Biology 24: 6488–6500. 15226448 10.1128/MCB.24.14.6488-6500.2004 PMC434242

[46] Rébé C, Cathelin S, Launay S, Filomenko R, Prévotat L, et al (2007) Caspase-8 prevents sustained activation of NF-*κ*B in monocytes undergoing macrophagic differentiation. Blood 109: 1442–1450. 17047155 10.1182/blood-2006-03-011585 PMC2492986

[47] Blazier A, Papin J (2012) Integration of expression data in genome-scale metabolic network reconstructions. Frontiers in Computational Physiology and Medicine 3: 299. 10.3389/fphys.2012.00299 PMC3429070 22934050

[48] Krawczyk C, Holowka T, Sun J, Blagih J, Amiel E, et al (2010) Toll-like receptor–induced changes in glycolytic metabolism regulate dendritic cell activation. Blood 115: 4742–4749. 20351312 10.1182/blood-2009-10-249540 PMC2890190

[49] Tannahill G, O'Neill L (2011) The emerging role of metabolic regulation in the functioning of Toll-like receptors and the NOD-like receptor Nlrp3. FEBS Letters 585: 1568–1572. 21565193 10.1016/j.febslet.2011.05.008

[50] Dinarello C (1996) Biologic basis for interleukin-1 in disease. Blood 87: 2095–2147. 8630372

[51] Mackman N, Brand K, Edgington T (1991) Lipopolysaccharide-mediated transcriptional activation of the human tissue factor gene in THP-1 monocytic cells requires both activator protein 1 and nuclear factor kappa B binding sites. The Journal of Experimental Medicine 174: 1517–1526. 1744583 10.1084/jem.174.6.1517 PMC2119026

[52] Richard G, Chang H, Cizelj I, Belta C, Julius A, et al.. (2011) Integration of large-scale metabolic, signaling, and gene regulatory networks with application to infection responses. 50th IEEE Conference on Decision and Control and European Control Conference (CDC-ECC),Orlando, FL, USA : 2227–2232.

[53] Lee J, Gianchandani E, Eddy J, Papin J (2008) Dynamic analysis of integrated signaling, metabolic, and regulatory networks. PLoS computational biology 4: e1000086. 18483615 10.1371/journal.pcbi.1000086 PMC2377155

[54] Karr J, Sanghvi J, Macklin D, Gutschow M, Jacobs J, et al (2012) A whole-cell computational model predicts phenotype from genotype. Cell 150: 389–401. 22817898 10.1016/j.cell.2012.05.044 PMC3413483

[55] Covert M, Xiao N, Chen T, Karr J (2008) Integrating metabolic, transcriptional regulatory and signal transduction models in Escherichia coli. Bioinformatics 24: 2044–2050. 18621757 10.1093/bioinformatics/btn352 PMC6702764

[56] Vardi L, Ruppin E, Sharan R (2012) A Linearized Constraint-Based Approach for Modeling Signaling Networks. Journal of Computational Biology 19: 232–240. 22300322 10.1089/cmb.2011.0277

[57] Thiele I, Fleming RMT, Que R, Bordbar A, Diep D, et al (2012) Multiscale modeling of metabolism and macromolecular synthesis in E. coli and its application to the evolution of codon usage. PLoS One in press. 10.1371/journal.pone.0045635 PMC3461016 23029152

[58] Thorleifsson S, Thiele I (2011) rBioNet: A COBRA toolbox extension for reconstructing highquality biochemical networks. Bioinformatics 27. 10.1093/bioinformatics/btr308 21596791

[59] Colicelli J (2004) Human RAS superfamily proteins and related GTPases. Science's STKE: signal transduction knowledge environment 2004: RE13. 10.1126/stke.2502004re13 PMC2828947 15367757

[60] Schumann R, Leong SR, Flaggs G, Gray P, Wright S, et al (1990) Structure and function of lipopolysaccharide binding protein. Science 249: 1429–1431. 2402637 10.1126/science.2402637

[61] Grube B, Cochane C, Ye R, Green C, McPhail M, et al (1994) Lipopolysaccharide binding protein expression in primary human hepatocytes and HepG2 hepatoma cells. Journal of Biological Chemistry 269: 8477–8482. 7510687

[62] Thomas C, Kapoor M, Sharma S, Bausinger H, Zyilan U, et al (2002) Evidence of a trimolecular complex involving LPS, LPS binding protein and soluble CD14 as an effector of LPS response. FEBS Letters 531: 184–188. 12417309 10.1016/s0014-5793(02)03499-3

[63] Warren P, Taylor D, Martini P, Jackson J, Bienkowska J (2007) PANP-a new method of gene detection on oligonucleotide expression arrays. In: Bioinformatics and Bioengineering, 2007. BIBE 2007. Proceedings of the 7th IEEE International Conference on. IEEE, pp. 108–115.

[64] Gentleman R, Carey V, Bates D, Bolstad B, Dettling M, et al (2004) Bioconductor: open software development for computational biology and bioinformatics. Genome biology 5: R80. 15461798 10.1186/gb-2004-5-10-r80 PMC545600

[65] Kostromins A, Stalidzans E (2012) Paint4Net: COBRA Toolbox extension for visualization of stoichiometric models of metabolism. BioSystems 10.1016/j.biosystems.2012.03.002 22446067

[66] Emig D, Salomonis N, Baumbach J, Lengauer T, Conklin B, et al (2010) AltAnalyze and Domain-Graph: analyzing and visualizing exon expression data. Nucleic Acids Research 38: W755–W762. 20513647 10.1093/nar/gkq405 PMC2896198

