# Supplementary material for: Contextualization Procedure and Modeling of Monocyte Specific TLR Signaling
Source: PLoS One. 2012 Dec 6;7(12):e49978. doi: 10.1371/journal.pone.0049978 (PMC3516512; doi:10.1371/journal.pone.0049978)
Supplement: File S1 — Workflow describing the generation of a cell-type specific network of TLR signaling in a step by step manner. (PDF) [file pone.0049978.s001.pdf]

Maike K. Aurich and Ines Thiele, Contextualization procedure and modeling of monocyte specific TLR signaling.

Workflow for the generation of a cell-type specific network of TLR-signaling

Requirements of software and packages:

- R (integrated suite of software facilities for data manipulation, calculation and graphical display, http://www.r-project.org/, [1]) and PANP package [2]
- Matlab (Mathworks, Inc)
- COBRA toolbox (http://opencobra.sourceforge.net/openCOBRA/Welcome.html, [3]) and a linear programming solver
- Paint4Net [4]

Step 1: Select data

- Download CEL files from e.g. Gene Expression Omnibus (GEO, http://www.ncbi.nlm.nih.gov/geo/, [5]).

Step 2: Generate P/A calls from gene expression data using R

- Use PANP package [2] in R for data processing and the generation of P/A calls.

Workflow in R:

```
>source(”http://bioconductor.org/biocLite.R”)biocLite(”panp”)

> setwd (e.g.’C:/...’)

> library(gcrma)

> Data < − ReadAffy() read data in working directory

> eset < − gcrma(Data)

>library(panp)

> PA < − pa.calls(eset, looseCutoff = 0.05,tightCutoff = 0.01, verbose = FALSE)

> myPcalls < − PA$Pcalls

> write.table(myPcalls, ”A_P_Calls”, append = FALSE, quote = TRUE,sep = ””,eol = ”\n” ,

   na = ”NA”,dec = ”.”, row.names = TRUE)

> myPvals < − PA$Pvals

> write.table(myPvals, ”A_P_vals”, append = FALSE, quote = TRUE,sep = ””,eol = ”\n”,

   na = ”NA”,dec = ”.”, row.names = TRUE)

> write.exprs(eset,file=”myresults.txt”)
```

This was the only analysis step done performed in R. From now on only use Matlab.

Step 3: Derive cell-type specific gene lists of absent genes from P/A calls at two different cutoffs

- Map Affymetrix IDs (A/P calls) to Entrez Gene IDs (ihsTLRv2.genes) (e.g using IDconverter http://idconverter.bioinfo.cnio.es/ [6]).
- If multiple Affymetrix IDs matched to one model gene, we used the Affymetrix IDs showing the highest mean expression intensity in the untreated group. For the generation of the monocyte model, we derived calls using Affymetrix IDs specified in (File S2, Table S14).
- Summarize calls for untreated replicates (to generate monocyte model) to receive one call (A or P) per ihsTLRv2 gene and cutoff ($p \leq 0.01$ and $p \leq 0.05$).
- Marginal calls are absent in the tight ($p \leq 0.01$) and present in the loose ($p \leq 0.05$) cutoff. If a gene received absent calls in the majority of replicates, call the gene absent.
- Lists of absent genes used for the generation of the monocyte draft models ($p \leq 0.01$ and $p \leq 0.05$) are provided in the supplementary information (File S2, Table S15).

Step 4: Generate of two draft models from lists of absent genes

- Use ihsTLRv2, lists of absent genes and the COBRA toolbox [3] function *deletemodelgenes* to generate two draftmodels.
- Set columns of constrained reactions in the draftmodel S matrix to zero.

Commands in Matlab:

```
[draftmodel,hasEffect,constrRxnNames,deletedGenes] = deleteModelGenes(model,geneList);

constrRxn= find(ismember(draftmodel,constrRxnNames));

    for i = 1 : length(constrRxn)

        draftmodel.S(:,constrRxn(i))=0;

    end
```

- The applied method relies entirely on the input (list of absent genes) and disables flux through any reaction associated with a deleted gene if no isozymes are associated. Gene expression data are known to be noisy, therefore it is important to find a suitable cutoff when using this method, and to perform manual curation afterwards.

Step 5: Find the best draft reconstruction with respect to cell-type

- Check if absent genes are absent at protein level in target cell-type using the Human Protein Atlas and decide based on this additional information, on the biologically most conclusive cutoff.

Step by step:

  - Find the set of genes absent in the tight ($p \leq 0.01$) and present in the loose ($p \leq 0.05$) cutoff.
  - Check for protein expression using the Human Protein Atlas (http://www.proteinatlas.org/) in the cell-type (herein, two monocytic leukemia cell lines (THP-1 and U-937)).
  - If the antibody yielded at least weak staining for the majority of tests in one cell sample, call the gene product present.
  - Use expression information and probability values from P/A calls (A_P_vals file generated by PANP) to define the most suitable cutoff.
  - Perhaps also take into consideration the number of blocked reactions, dead ends and input receptor covered by either draftmodel.

Commands in Matlab:

```
[minFlux,maxFlux] = fluxVariability(draftmodel,0);

Flux = [minFlux maxFlux];

        for i = 1 : length(Flux)

            x = length (find (abs(Flux(i,:))<=10e-10==0))==2;

            Blockedrxns(i,1) = x;

        end

        for i = 1:length(draftmodel.mets)

            MetConn(i,1)=length(find(draftmodel.S(i,:)));

        end

MetConnCompare1 = sort(MetConnTLR,'descend');

deadends(1,1) = length(find(MetConnCompare1(:,1)==1));
```

- Decide on a suitable cutoff.
- For the monocyte, we decided to continue with the draftmodel generated using data from the loose ($p \leq 0.05$) cutoff.
- If necessary, repeat draft model generation based on absent gene set of the newly defined cutoff (Step 4).

Step 6: Curate draftmodel: complete disconnected output pathways

- Check using Flux variability analysis (FVA) if the draftmodel can produce all outputs known to be produced in the cell-type.

Command in Matlab:

```
[minFlux,maxFlux] = fluxVariability(model,0);
```

  - Monocytes produce all six ihsTLRv2 outputs. However, the monocyte draftmodel was not able to produce e.g. NF-κB.

- Search for candidate genes to complete disconnected output pathways using the pathway illustration tool, Paint4Net [4]:

  - First, identify a list of reactions involved in the signaling pathway towards NF-κB using ihsTLRv2, which contains the complete pathways, as reference. Choose a wide radius such that *involvedRxns* contains the complete pathway from an input to the specified output to capture the entire pathway.

Command in Matlab:

```
[involvedRxns,involvedMets,deadEnds]=draw_by_met(model,metAbbr,drawMap,...

    radius,direction,excludeMets,flux);
```

- Do the same for the draftmodel and the disconnected output pathways.
- Compare the resulting list of participating reactions to reveal the missing links in the draftmodel. Find the genes associated with these reactions. These are the candidate genes.
- Generate draft models, while reincorporating one candidate gene at a time and check the impact on output production.
- Candidate genes identified using this approach and output fluxes derived after reincorporation of candidate genes was added to the supplementary information (File S2, Table S6).

Step 7: Curate draftmodel: Curate model based on cell-type specific literature

- Search for literature evidence of absent genes being expressed in the specific cell-type.
  - We only considered genes, which were absent in the monocyte draftmodel, while isoforms of already captured genes were ignored.
  - During manual curation, 14 genes were reintroduced to the ihsMonoTLR model based on literature support (File S2, Table S7).

Step 8: Derive curated cell-type model

- Update gene list by removing curated genes from list of absent genes at defined cutoff ($p \leq 0.05$).
- Generate final cell-type model based on deletion of curated gene list (Step 4).
- The absent gene list used for the generation of the final monocyte model is provided in the supplementary information (File S2, Table S15).
- Use COBRA function *extractSubNetwork* and the set of reactions that remained unconstrained during model generation (*constrRxnNames* output of *deletemodelgenes* provides the list of constrained reactions).

Command in Matlab:

```
subModel = extractSubNetwork(model,rxnNames)
```

Step 9: Tailor cell-type model condition specific

- Find list of cell-type model genes absent in the specific condition (e.g. LPS treatment of monocytes).
- Repeat Step 4 using the final cell-type model as model and the set of absent genes.
- In case of the monocyte model two genes (Entrez Gene ID: 246330 and 10333) were deleted in order to derive the LPS-stimulation specific monocyte network (hMonoTLR_LPS).

References

1. Gentleman R, Carey V, Bates D, Bolstad B, Dettling M, et al. (2004) Bioconductor: open software development for computational biology and bioinformatics. Genome biology 5: R80.

2. Warren P, Taylor D, Martini P, Jackson J, Bienkowska J (2007) PANP-a new method of gene detection on oligonucleotide expression arrays. In: Bioinformatics and Bioengineering, 2007. BIBE 2007. Proceedings of the 7th IEEE International Conference on. IEEE, pp. 108–115.

3. Schellenberger J, Que R, Fleming R, Thiele I, Orth J, et al. (2011) Quantitative prediction of cellular metabolism with constraint-based models: the COBRA Toolbox v2.0. Nature Protocols 6: 1290–1307.

4. Kostromins A, Stalidzans E (2012) Paint4Net: COBRA Toolbox extension for visualization of stoichiometric models of metabolism. BioSystems .

5. Edgar R, Domrachev M, Lash A (2002) Gene Expression Omnibus: NCBI gene expression and hybridization array data repository. Nucleic acids research 30: 207–210.

6. Alibés A, Yankilevich P, et al. (2007) IDconverter and IDClight: Conversion and annotation of gene and protein IDs. BMC bioinformatics 8: 9.
