# Supplementary material for: Contextualization Procedure and Modeling of Monocyte Specific TLR Signaling
Source: PLoS One. 2012 Dec 6;7(12):e49978. doi: 10.1371/journal.pone.0049978 (PMC3516512; doi:10.1371/journal.pone.0049978)
Supplement: File S3 — Sensitivity analysis figures for all input/output relationships (Figures S1, S2, S3, S4, S5, S6, S7, S8, S9, S10, S11, S12, S13). (PDF) [file pone.0049978.s003.pdf]

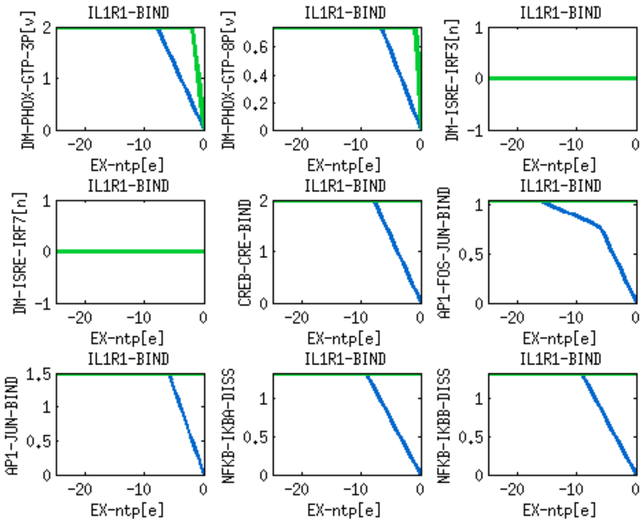

Figure 1. Sensitivity analysis

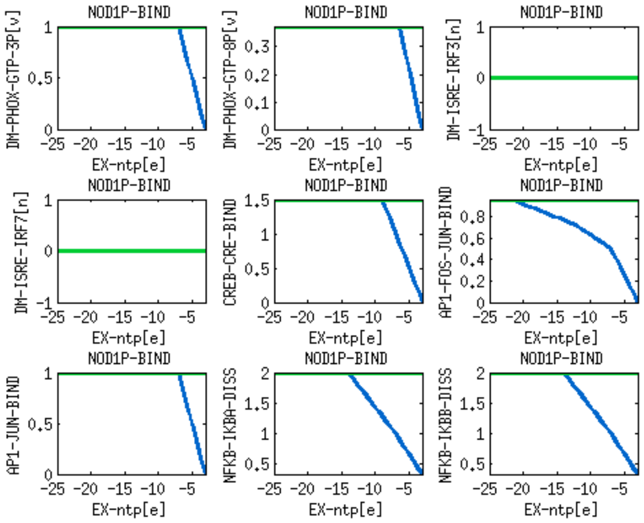

Figure 2. Sensitivity analysis

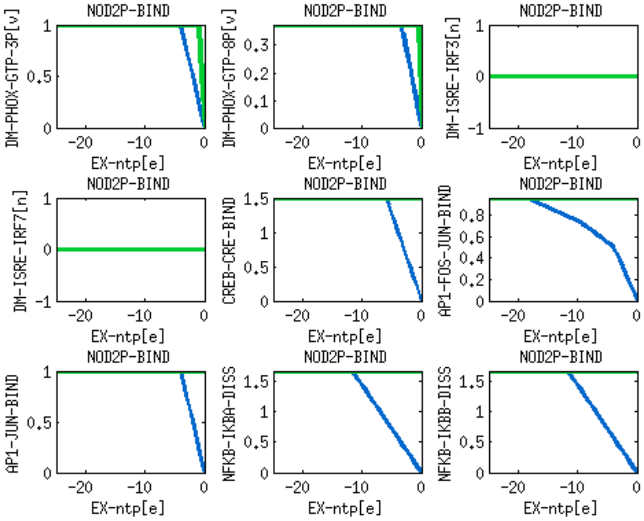

Figure 3. Sensitivity analysis

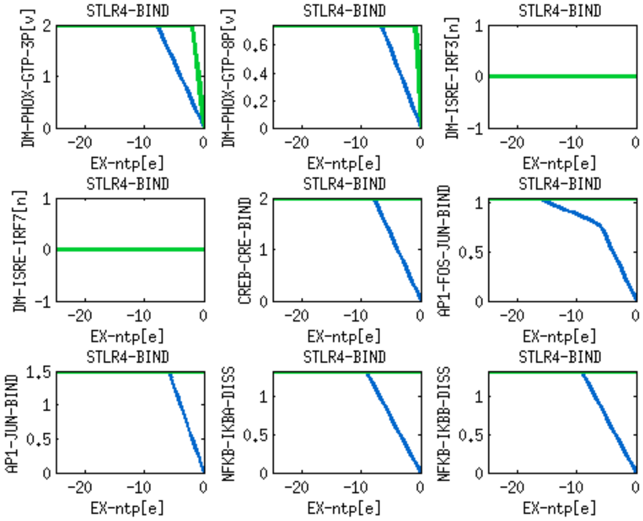

Figure 4. Sensitivity analysis

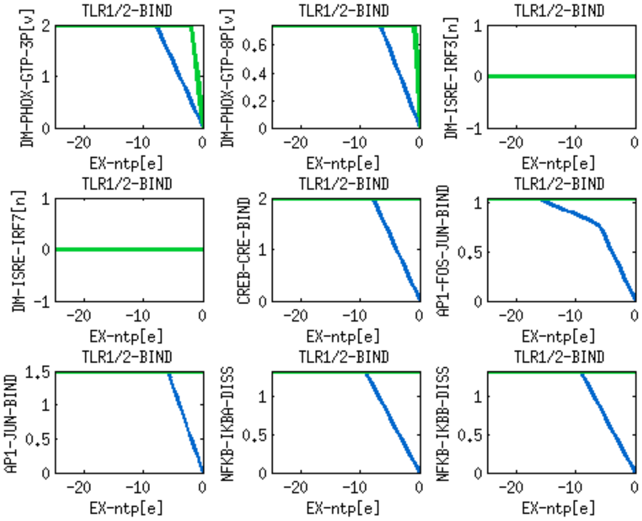

Figure 5. Sensitivity analysis

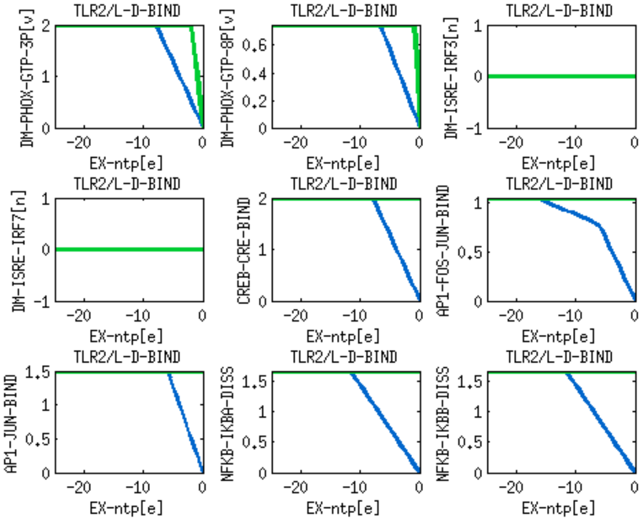

Figure 6. Sensitivity analysis

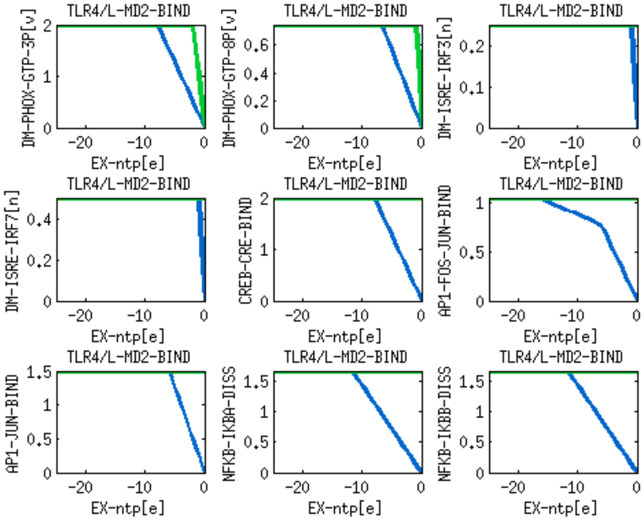

Figure 7. Sensitivity analysis

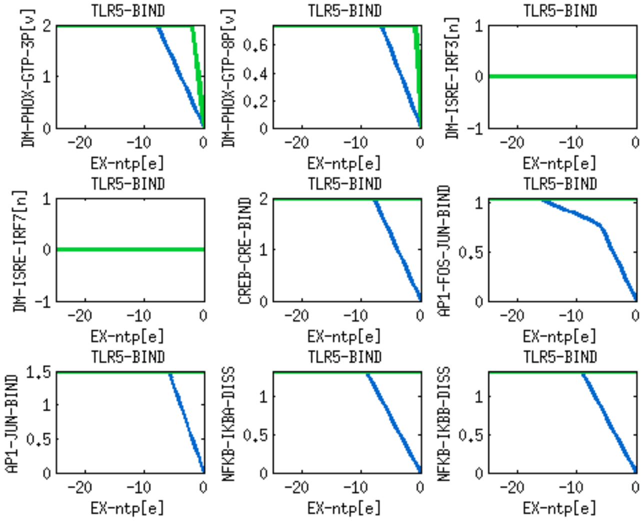

Figure 8. Sensitivity analysis

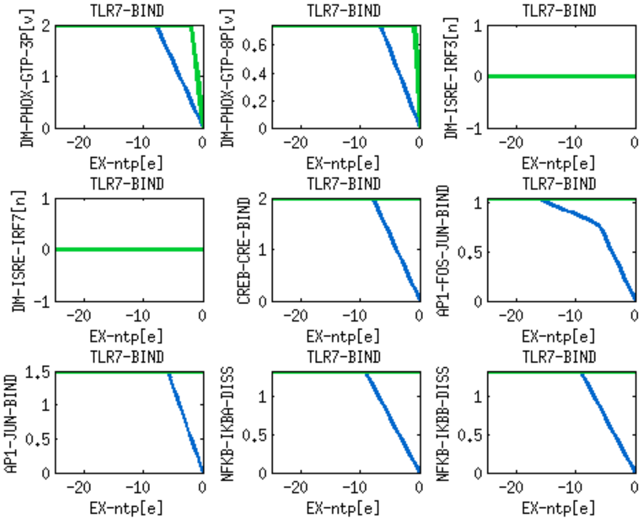

Figure 9. Sensitivity analysis

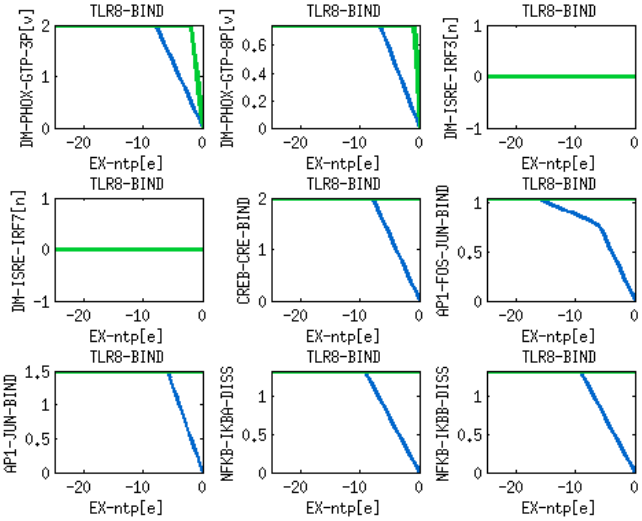

Figure 10. Sensitivity analysis

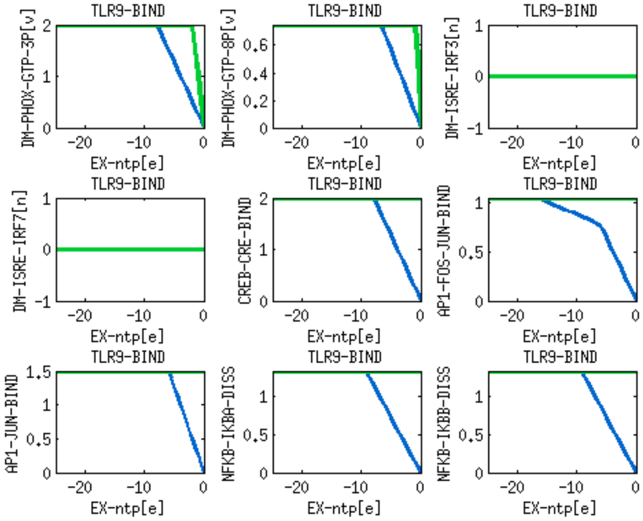

Figure 11. Sensitivity analysis

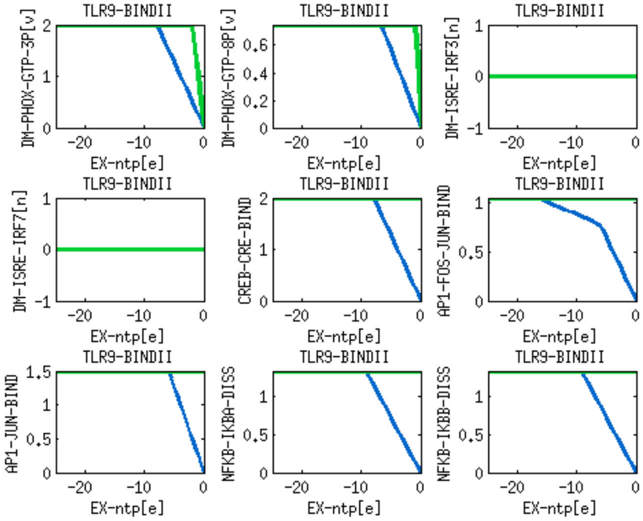

Figure 12. Sensitivity analysis

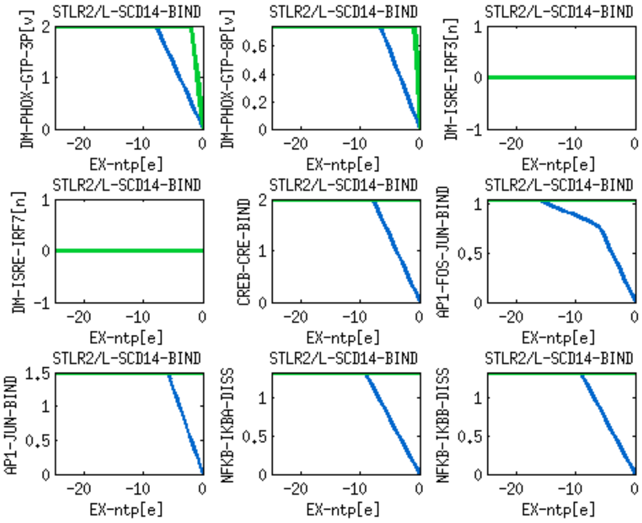

Figure 13. Sensitivity analysis
